# Patient experiences in primary care do not differ according to rurality: a cross-sectional study

**DOI:** 10.1186/s12875-024-02397-2

**Published:** 2024-04-25

**Authors:** Makoto Kaneko, Hironori Yamada, Tadao Okada

**Affiliations:** 1 Department of Health Data Science, Yokohama City University, 22-2, Seto, Kanazawa-ku, Yokohama, Kanagawa 236-0027 Japan; 2 Department of Family and Community Medicine, Hamamatsu University School of Medicine, 1-20-1, Handayama, Higashi-ku, Hamamatsu, 431-3192 Japan; 3 Department of Family Medicine, Graduate School, Tokyo Medical and Dental University, Tokyo, 113-8519 Japan; 4 Department of Family Medicine, Kameda Family Clinic Tateyama, Chiba, Japan

**Keywords:** Patient experiences, Primary care, Rural health, Rurality

## Abstract

**Background:**

Living in rural areas is a major contributor of health inequity. Tackling health inequity is important for primary care physicians. Therefore, it is important to compare the quality of primary care between rural and urban areas. To the best of our knowledge, this is the first study to examine the association between rurality and patient experience (PX) in Japan using validated measures.

**Methods:**

This cross-sectional study was conducted using online surveys. Participants were selected using a stratified random sample based on sex and age. The Japanese version of the Person-Centered Primary Care Measure (PCPCM) was used as an indicator of PX. We used the Rurality Index for Japan (RIJ) to measure rurality. Furthermore, we used multivariate linear regression analysis to examine the relationship between the RIJ and PCPCM after adjusting for confounders.

**Results:**

Of the 1112 eligible participants, 800 responded to the survey (response rate:71.9%). The mean PCPCM scores were 2.46 (standard deviation: 0.73) and median RIJ was 15 (interquartile range: 6–33). The crude and adjusted coefficients of rurality were − 0.02 (− 0.006–0.001, *p* = 0.114) and − 0.02 (− 0.005–0.001), respectively, demonstrating that rurality was not significantly associated with the total PCPCM score. Subgroup analyses were similar to the main analyses.

**Conclusion:**

We found that PX in primary care did not differ by rurality in the general Japanese population.

## Background

Health inequity is a global issue and part of the Quintuple Aim which was proposed by Nundy et al. in 2022 [1]. This concept adds health equity to the quadruple aim of patient experience (PX), population health, cost, and provider satisfaction [1, 2]. Health inequity occurs according to sex, ethnicity, and socioeconomic status and affects multiple aspects of individual and population health, such as behavioural risk factors and mortality [3].

Primary care physicians (PCPs) play an important role in overcoming health inequity [4–6]. They are located in a unique position on the front line of the healthcare system to address health inequity [5]; hence, they can detect unjust health outcomes [5], advocate for patients, and collaborate with stakeholders [4]. The supply of PCPs is associated with reductions in racial, socioeconomic, and geographical disparities [6].

Living in rural areas is one of the contributors of health inequity [7] and is generally associated with higher rates of unemployment, lower educational attainment, and less access to healthcare and social services [7]. Additionally, people in rural areas may experience barriers to travel for secondary care, have limited healthy food options, and have a lack of access to technology including broadband [7]. Such an environment around patients affects their healthcare outcomes [8]. PCPs have the knowledge, skills and experience to address this issue [4].

Therefore, improving the quality of primary care is essential for addressing health inequity in rural areas. To measure the quality of primary care, PX is indispensable [9] and is a part of the Quintuple Aim [1]. Although some studies have compared PX in rural and urban areas, the results are inconsistent. Studies in China and Scotland reported that PX in primary care were better in rural areas than in their urban counterparts [10, 11]. However, a study in the US revealed that PX in rural areas were worse than that in urban areas [12]. Moreover, to the best of our knowledge, the measures of PX in these studies were not validated [10–12] and did not cover essential core domains of primary care, such as: first contact, longitudinality, comprehensiveness, and coordination of care [13].

Hence, this study aimed to examine the association between rurality and PX using validated measures as investigating the disparity in PX between rural and urban areas in primary care settings is important for improving healthcare in rural areas.

## Methods

### Design

This was a cross-sectional study using an online survey. The survey was based on the previous literature [14].

### Setting and participants

Participants were selected from an online survey company, RJC Research, which has a pool of survey participants, including people from across Japan. In this study, the company recruited 1000 potential participants from the pool using a stratified random sample based on sex and age. The questionnaire was distributed to participants via email. The study included participants aged from 20 to 74 years because the Japanese version of the Person-Centred Primary Care Measure (PCPCM), the quality indicator of primary care used in the study, targeted this age group [14].

### Measures

#### Outcome variable

The Japanese version of the PCPCM was used to assess PX in this study [14]. The Japanese version of the PCPCM has sufficient reliability and validity [14]. The PCPCM was developed in the US in 2019 to assess PX in primary care settings [15] and has been translated into 28 languages [16]. This measure assesses 11 core domains of primary care using 11 items: accessibility, comprehensiveness, integration, coordination, relationship, continuity, advocacy, family context, community context, goal-oriented care, and health promotion. Each domain is described to be between 1 and 4, with 4 being the highest score. The total score is the average score of the 11 domains, which ranges between 1 and 4. Because the role of PCPs is ambiguous in Japan [14], the Japanese version of the PCPCM uses the question, “Is there a medical facility to whom you usually go if you are sick or need advice about your health?” to identify whether participants had a usual source of care (USC) [14, 17].

#### Exposure variable

The Rurality Index for Japan (RIJ) was employed as an indicator of rurality [18]. The RIJ consists of four factors: population density, distance to secondary or tertiary care hospitals, remote islands, and heavy snow areas [18]. The score is calculated by adding each factor with weight and describes rurality from 1 to 100 for all zip codes in Japan (with 100 being the most rural) [18]. The RIJ showed good validity in the validation study [18].

#### Covariates

Covariates were determined by known associations with rurality and PX based on previous literature [10–12]. We included age (categorical variable; 5-year age group), sex (binary variable), education status (categorical variable: less than high school, high school, junior college, and more than or equal to college), annual household income (categorical variable: < 3, 3–5.99, 6–8.99, 9–11.99, 12–14.99, and ≥ 15 million JPY), self-rated health (categorical variable; excellent, good, neutral, poor, and very poor), marital status (binary variable: married or not), types of USC (binary variable: clinic or hospital), and the number of chronic health problems (categorical variable: 1–20). Data on chronic health problems, including hypertension, depression/anxiety, chronic musculoskeletal conditions causing pain or limitation, arthritis/rheumatoid arthritis, osteoporosis, chronic respiratory disease (asthma, chronic obstructive pulmonary disease, or chronic bronchitis), cardiovascular disease, heart failure, stroke/transient ischaemic attack, gastric issues, colon problems, chronic hepatitis, diabetes, thyroid disorder, any cancer, kidney disease/failure, chronic urinary problems, dementia/Alzheimer’s disease, hyperlipidaemia, and obesity was also evaluated [19]. All data were collected from the participants through the survey.

#### Statistical analysis

Categorical variables are presented as numbers and proportions. Continuous variables are described as medians and interquartile ranges. We used the RIJ and PCPCM as continuous variables, and the relationship between the RIJ and PCPCM was visualised using a scatter plot. We performed a multivariable linear regression analysis to examine the relationship between the RIJ and PCPCM. As a sensitivity analysis, we conducted a subgroup analysis by age (< 65 and ≥ 65 years) and location of USC (clinic or hospital) to examine the impact of age/location of USC on PX among the different groups. For the sample size, we input eight factors into the model; therefore, approximately 100 participants were required for the study. In a previous study which examined PX by PCPCM in Japan, 50–60% of the participants had a USC [14]; moreover, the response rate was 20–30%. Hence, we targeted approximately 1000 people. All statistical analyses were performed using Stata Statistical Software Release 15 (StataCorp LLC, College Station, TX, USA).

#### Ethical approval

This study was approved by the Ethics Committee of Yokohama City University (approval number: F220900057). All the participants provided written informed consent to participate in the study.

## Results

**Fig. 1:**
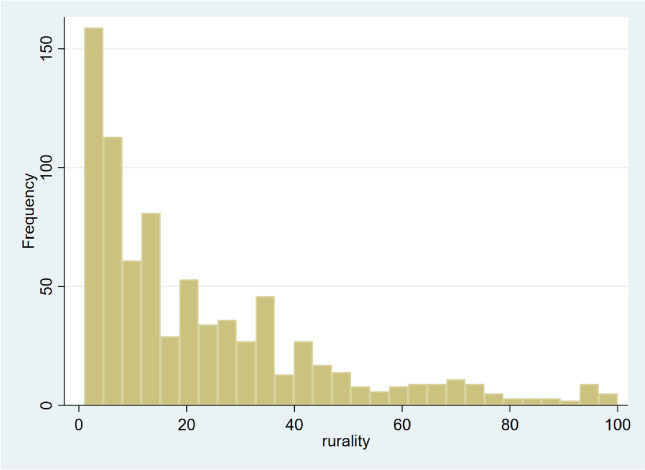
Histogram of the rurality of the participants

**Fig. 2:**
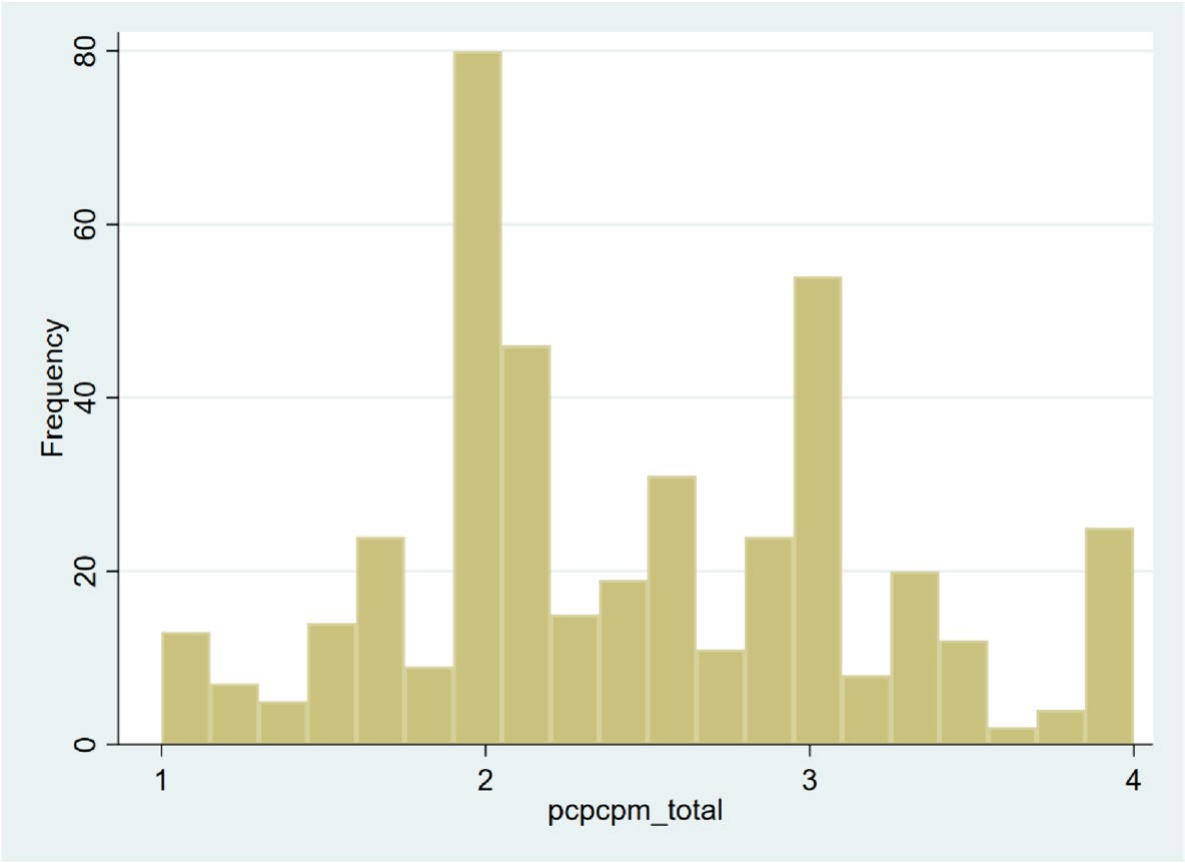
Histogram of the total PCPCM score

**Fig. 3:**
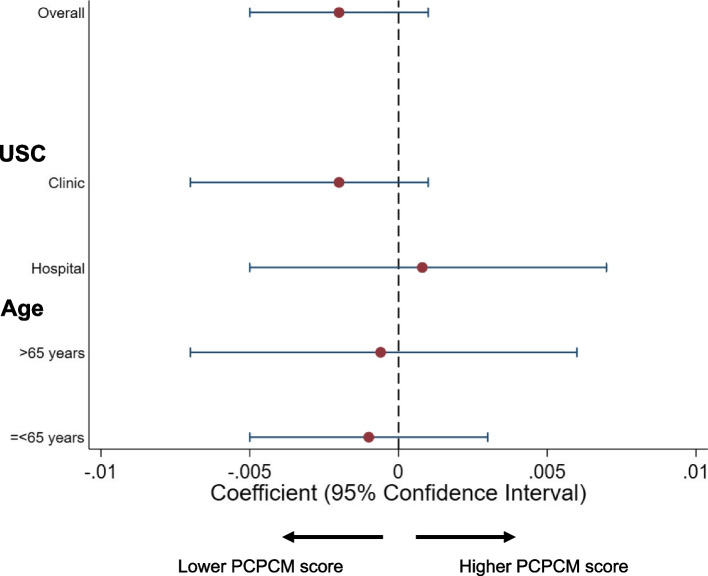
Forest plot of the overall results and subgroup analyses

We offered the opportunity to 1112 participants to participate in the study and 800 responded (response rate: 71.9%). No variables were missing. The number of participants who had a USC was 423 (52.9%): 297 (70.2%) used a clinic and 126 (29.8%) used a hospital. The characteristics of the participants with and without a USC are shown in Table 1. Figure 1 shows a histogram of the rurality of the participants, and Fig. 2 shows a histogram of the total PCPCM score. The mean PCPCM score was 2.46 (standard deviation [SD]:0.73). The mean scores for each item in the PCPCM were: accessibility, 2.66 (SD: 0.83); comprehensiveness, 2.65 (SD: 0.84); integration, 2.50 (SD: 0.87); coordination, 2.44 (SD: 0.86); relationship, 2.71 (SD: 0.86); continuity, 2.28 (SD: 0.90); advocacy, 2.45 (SD: 0.86); family context, 2.24 (SD: 0.92); community context, 2.23 (SD: 0.82); goal-oriented care, 2.43 (SD: 0.87); and health promotion, 2.51 (SD: 0.86). The multivariate regression analysis demonstrated that rurality was not significantly associated with the total PCPCM score. The crude and adjusted coefficients of rurality were − 0.02 (− 0.006–0.001, *p* = 0.114) and − 0.02 (− 0.005–0.001), respectively. The coefficients of each variable are listed in Table 2. Crude and adjusted coefficients of RIJ for PCPCM are described in Table 3. Figure 3 shows a forest plot of the results of the main and subgroup analyses. The trends in the results are similar to those in the main analysis.

**Table 1:** Participant characteristics. (*n* = 800)

	Overall *n* (%)	With USC *n* = 423 *n* (%)	Without USC *n* = 377 *n* (%)
Sex			
Male	399 (49.8)	196 (46.3)	203 (53.8)
Female	400 (50.0)	227 (53.7)	173 (45.9)
Others	1 (0.1)	0 (0)	1 (0.3)
Age (years)			
20–24	41 (5.1)	19 (4.5)	22 (5.8)
25–29	86 (10.8)	31 (7.3)	55 (14.6)
30–34	51 (6.4)	17 (4.0)	34 (9.0)
35–39	75 (9.4)	34 (8.0)	41 (10.9)
40–44	85 (10.6)	38 (9.0)	47 (12.5)
45–49	81 (10.1)	49 (11.6)	32 (8.5)
50–54	78 (9.8)	42 (9.9)	36 (9.5)
55–59	74 (9.3)	36 (8.5)	38 (10.1)
60–64	83 (10.4)	49 (11.6)	34 (9.0)
65–69	61 (7.6)	51 (12.1)	10 (2.7)
70–74	85 (10.6)	57 (13.5)	28 (7.4)
Education			
< High school	13 (1.6)	7 (1.7)	6 (1.6)
High school	238 (29.8)	127 (30.0)	111 (29.4)
Junior college	174 (21.8)	96 (22.7)	78 (20.7)
≥ College	375 (46.9)	193 (45.6)	182 (48.3)
Annual household income (million JPY)			
< 3.00 (≒27,000 US dollar)	166 (20.8)	78 (18.4)	88 (23.3)
3.00–5.99	208 (26.0)	128 (30.3)	80 (21.2)
6.00–8.99	124 (15.5)	65 (15.4)	59 (15.6)
9.00–11.99	77 (9.6)	39 (9.2)	38 (10.1)
12.00–14.99	35 (4.4)	17 (4.0)	18 (4.8)
≥ 15.00	24 (3.0)	16 (3.8)	8 (2.1)
Unknown	166 (20.8)	80 (18.9)	86 (22.8)
Self-rated health status			
Excellent	39 (14.3)	20 (4.7)	19 (5.0)
Good	110 (13.8)	57 (13.5)	53 (14.1)
Neutral	228 (28.5)	112 (26.5)	116 (30.8)
Poor	309 (38.6)	165 (39.0)	144 (38.2)
Very poor	114 (14.3)	69 (16.3)	45 (11.9)
Marital status			
Married	395 (49.4)	237 (56.0)	158 (41.9)
Divorced	81 (10.1)	43 (10.2)	38 (10.1)
Never married	304 (38.0)	131 (31.0)	173 (45.9)
Widowed	18 (2.3)	11 (2.6)	7 (1.9)
Others	2 (0.3)	1 (0.2)	1 (0.3)
Employment status			
Full-time employee	345 (43.1)	156 (36.9)	189 (50.1)
Part-time employee	155 (19.4)	89 (21.0)	66 (17.5)
Self-employee	47 (5.9)	27 (6.4)	20 (5.3)
Unemployed	236 (29.5)	144 (34.0)	92 (24.4)
Others	17 (2.1)	7 (1.7)	10 (2.7)
Number of chronic conditions			
0	472 (59.0)	171 (40.4)	301 (79.8)
1	202 (25.3)	148 (35.0)	54 (14.3)
2	78 (9.8)	65 (15.4)	13 (3.4)
3	26 (3.3)	22 (5.2)	4 (1.1)
4	15 (1.9)	11 (2.6)	4 (1.1)
5	4 (0.5)	3 (0.7)	1 (0.3)
6	1 (0.1)	1 (0.2)	0 (0)
7	2 (0.3)	2 (0.5)	0(0)
Types of USC			
Clinic	297 (70.2)	297 (70.2)	
Hospital	126 (29.8)	126 (29.8)	
Median of RIJ			
(IQR)	15 (6–33)	15 (6–34)	14 (6–32)

*USC* usual source of care, *IQR* interquartile range, *RIJ* Rurality Index for Japan

**Table 2:** Association between PCPCM and each variable: the results of the multivariable linear regression analysis

(*n* = 423)	Coefficient (95% CI, *p*-value)
RIJ	− 0.002 (− 0.005–0.001, *p* = 0.22)
Sex	
Male	Reference
Female	− 0.06 (− 0.22–0.09, *p* = 0.43)
Age (year)	
20–24	Reference
25–29	−0.18 (− 0.60–0.23, *p* = 0.067)
30–34	0.08 (− 0.40–0.55, *p* = 0.75)
35–39	− 0.14 (− 0.55–0.27, *p* = 0.50)
40–44	− 0.10 (− 0.50–0.31, *p* = 0.64)
45–49	− 0.08 (− 0.47–0.32, *p* = 0.83)
50–54	−0.014 (− 0.41–0.38, *p* = 0.95)
55–59	− 0.04 (− 0.45–0.36, p = 0.83)
60–64	−0.14 (− 0.54–0.25, *p* = 0.48)
65–69	−0.12 (− 0.53–0.29, *p* = 0.56)
70–74	−0.15 (− 0.57- -0.26, *p* = 0.46)
Education	
< High school	Reference
High school	0.48 (−0.07–1.02 *p* = 0.09)
Junior college	0.59 (0.04–.15, *p* = 0.036)
≥ College	0.42 (−0.14–0.96, *p* = 0.14)
Annual household income (million JPY)	
< 3.00 (≒27,000 US dollar)	Reference
3.00–5.99	0.05 (−0.15–0.26, *p* = 0.62)
6.00–8.99	−0.05 (− 0.30–0.21, *p* = 0.72)
9.00–11.99	0.15 (− 0.14–0.44, *p* = 0.30)
12.00–14.99	0.25 (− 0.14–0.63, *p* = 0.21)
≥ 15.00	0.38 (− 0.03–0.78, *p* = 0.07)
Unknown	−0.08 (− 0.3–0.15, *p* = 0.51)
Self-rated health status	
Very poor	Reference
Poor	0.11 (−0.26–0.49, *p* = 0.55)
Neutral	−0.11 (− 0.46–0.24, *p* = 0.53)
Good	− 0.43 (− 0.78- -0.08, *p* = 0.016)
Excellent	−0.62 (−1.01- -0.24, *p* = 0.002)
Marital status	
Married	Reference
Not married	0.66 (0.30–1.01, *p* < 0.001)
Types of USC	
Hospital	Reference
Clinic	0.06 (−0.09- -.21, *p* = 0.45)
Number of chronic conditions	
0	Reference
1	0.20 (0.04–0.37, *p* = 0.017)
2	0.23 (0.01–0.49, *p* = 0.04)
3	0.32 (−0.006–0.65, *p* = 0.054)
4	0.41 (−0.04–0.86)
5	1.27 (0.44–2.10, *p* = 0.003)
6	0.03 (−1.39–1.44, *p* = 0.97)
7	0.12 (−0.89–1.15, *p* = 0.81)

*USC* usual source of care, *RIJ* Rurality Index for Japan

**Table 3:** Crude and adjusted coefficients of RIJ for PCPCM

	Crude coefficient (95% CI)	Adjusted coefficient* (95% CI)
Main analysis		
All participants (n = 423)	−0.002 (− 0.006–0.001)	−0.002 (− 0.005–0.001)
Subgroup analysis		
Type of USC: Clinic (*n* = 297)	−0.004 (− 0.007–0.0001)	−0.002 (− 0.007- -0.001)
Type of USC: Hospital (*n* = 126)	0.00003 (− 0.005–0.006)	0.0008 (− 0.005–0.007)
≥65 (*n* = 108)	−0.0001 (− 0.006–0.005)	−0.0006 (− 0.007–0.006)
65< (*n* = 315)	−0.003 (− 0.007–0.0003)	−0.001 (− 0.005–0.003)

*RIJ* Rurality Index for Japan, *USC* usual source of care, *CI* confidence interval

*Adjusted for age, sex, education status, annual household income, self-rated health, marital status, types of USC, and the number of chronic health problems

## Discussion

This study demonstrates that PX in primary care do not differ according to rurality in the general Japanese population. After adjusting for covariates, rurality was not found to be significantly associated with PX; hence, this may indicate that primary care is provided without inequity even in rural areas of Japan.

Universal healthcare coverage and public assistance for low-income populations would have ensured access medical care [20] and been the reason for these results. Furthermore, although Japan has many remote islands and 58% comprises “depopulated areas” [21], distance to healthcare facilities can not be long compared with large countries such as China or the US. The area of these countries is around 26 times that of Japan. Therefore, the difference in healthcare access by rurality would be relatively small in comparison to these countries. Moreover, Japan has a university which has gathered students from across Japan and has fostered and placed physicians to rural area as their tuition repayment program for 50 years [22]. These graduates in public clinics or hospitals might contribute to maintaining rural primary care in Japan. In terms of PX in primary care, health inequity is currently not obvious; however, this may gradually increase because rural people tend to move from rural to urban areas and the population in urban areas is increasing [23]. Therefore, prospectively, the rural-urban healthcare disparity may have a greater impact.

The association between rurality and PX has been inconsistent in previous studies; moreover, the measures of PX used in these studies were not validated. Zhao et al. reported PX in urban residents was worse than that in rural residents [10]. In this study, the authors used the question “: “What score will you give to healthcare services (0–100)?” to assess PX. Iqbal et al. demonstrated the relationship between the quality of primary care and rurality is necessarily not linear [11]. In their study, patient satisfaction in suburban areas was lower than that in urban and rural areas [11]. The questions employed to measure PX in the study were: ‘I was listened to’, ‘I was given enough time’, ‘I was treated with compassion and understanding’, ‘I was given the opportunity to involve the people that matter to me’, ‘I understood the information I was given’, ‘I was in control of my treatment/care’, ‘I knew the healthcare professional well’, and ‘my treatment/care was well coordinated’. [11] In the US, Medicare beneficiaries reported lower satisfaction with care than their urban counterpart among people aged 65 years and over [12]. This study used a Likert scale from 1 to 4 (very dissatisfied, dissatisfied, satisfied, and very satisfied) for the assessment of patient satisfaction [12]. These questions were not validated as a measure of PX or patient satisfaction in any of these studies. In a review article which assessed the quality of care in home care settings, the results of PX in rural and urban settings were inconsistent [24]. Our study utilised validated and comprehensive measures of PX in primary care [14], providing meaningful comparisons regarding rural and urban primary care quality.

In terms of an index of rurality, the previous study in China used a dichotomous category (rural/urban) and the definition is ambiguous [10]. The other study in the US employed a three-tiered category (metropolitan county, rural micropolitan county and rural noncore county) based on the population [12]. These classifications did not describe the gradation between rural and urban. The RIJ which was used in the study is continuous variable with the value between 1 and 100 instead of categorical variable used in Xhao’s study [12], which leads to better detection of fine difference. Also, RIJ is validated by meaningful correlation with other indicators such as physician distribution and average life expectancy [18]. Therefore, our study holds higher validity than the previous studies [18]. This also contributed to a relevant comparison.

### Study strengths

To the best of our knowledge, this was the first study to examine the association between rurality and PX in primary care facilities in Japan. Furthermore, this study employed validated measures of rurality and PX. In addition, stratified random sampling from a pool of potential participants across Japan was used to mitigate selection bias. This study assessed the comprehensive quality of primary care using the PCPCM.

### Study limitations

This study had some limitations. First, owing to the cross-sectional nature of the study, we could not determine the causality between rurality and PX. Second, the study participants mainly lived in areas with low RIJ scores. The median of RIJ score was 15 in the study. Although the cut-off or rural-urban definition of the RIJ was not determined, many cities with RIJ 15 were sub-urban area around Tokyo or prefectural capital cities. This might be explained by depopulation in rural areas. However, it was possible that members of the survey participant pool were biased toward urban areas.

## Conclusion

We found that PX in primary care did not differ by rurality in the general Japanese population.

## Data Availability

The datasets generated and analyzed in the current study are not publicly available as we did not receive written informed consent for data sharing.

## Abbreviations

PX: patient experience
PCP: primary care physician
PCPCM: person-centered primary care measure
RIJ: rurality index for Japan
USC: Usual Source of Care
