# Correction: Patient experiences in primary care do not differ according to rurality: a cross-sectional study

**DOI:** 10.1186/s12875-025-02752-x

**Published:** 2025-02-22

**Authors:** Makoto Kaneko, Hironori Yamada, Tadao Okada

**Affiliations:** 1 Department of Health Data Science, Yokohama City University, 22-2, Seto, Kanazawa-ku, Yokohama, Kanagawa 236-0027 Japan; 2 Department of Family and Community Medicine, Hamamatsu University School of Medicine, 1-20-1, Handayama, Higashi-ku, Hamamatsu, 431-3192 Japan; 3 Department of Family Medicine, Graduate School, Tokyo Medical and Dental University, Tokyo, 113-8519 Japan; 4 Department of Family Medicine, Kameda Family Clinic Tateyama, Chiba, Japan

**Correction:** ***BMC Prim. Care*** **25, 132 (2024)**

10.1186/s12875-024-02397-2

Following publication of the original article [1], the authors reported that the name of ‘Tadao Okada’ was incorrectly presented as ‘Tadao Oakada’ in the author group.

The original article [1] has been corrected.
